# Development and applicability of a dignity‐centred palliative care programme for people with idiopathic pulmonary fibrosis: A qualitative‐driven mixed methods study

**DOI:** 10.1002/nop2.1274

**Published:** 2022-06-20

**Authors:** Yasuko Igai, Sarah E. Porter

**Affiliations:** ^1^ Graduate School of Nursing Science St. Luke's International University Tokyo Japan; ^2^ Oregon Health & Science University School of Nursing Portland USA

**Keywords:** dignity, dignity‐centered care, idiopathic pulmonary fibrosis, interstitial lung disease, nursing, palliative care

## Abstract

**Aims:**

This study evaluated the acceptability of a dignity‐centred palliative care programme for people with idiopathic pulmonary fibrosis by converging perceptions of living with idiopathic pulmonary fibrosis qualitative data and quantitative data.

**Design:**

The qualitative‐driven mixed methods research addressed the study aim by using a convergent design. This single arm, non‐randomized study used purposive sampling.

**Methods:**

Interviews with 12 stable outpatients with IPF provided qualitative data. Their quantitative data were from six scales: self‐esteem, health‐related quality of life, anxiety, depression, dyspnoea, cough and programme satisfaction. Intervention was three educational modules: symptom management, enhancing daily activities and life reviews.

**Results:**

Semi‐structured interviews yielded eight categories. Self‐esteem was not statistically significantly changed. Dyspnoea symptoms improved significantly. Participants (*n* = 9) holding positive attitudes for living with idiopathic pulmonary fibrosis, had improved lifestyle behaviour and improved or maintained self‐esteem. The meta‐inference regarding idiopathic pulmonary fibrosis perceptions were related to changes in self‐esteem.

## 1 INTRODUCTION

Idiopathic pulmonary fibrosis (IPF) is a rare, intractable and fatal disease (Mei et al., 2022). Maher et al. (2021) target literature review covering 2009 to 2020 found 22 studies covering 12 countries. The adjusted incidence was from .09–1.30 per 10,000. The adjusted prevalence was from 3–4.51 per 10,000. The lowest incidence and prevalence were found in the Asia‐Pacific countries with the exception of South Korea with the highest of all countries. Globally, the incidence is increasing (Maher et al., 2021; Strongman et al., 2018). Japanese people with IPF have a faster illness progressive course; deaths due to acute exacerbations are 40%, although, in other countries 25% died due to respiratory failure (Natsuizaka et al., 2014).

IPF is characterized by increasing dyspnoea and cough leading to complete dependence on oxygen therapy (Japan Respiratory Society, 2016). Even after symptoms emerge, receiving a correct diagnosis may involve delays (van der Sar et al., 2021) changing doctors, and waiting for diagnostic results (Schoenheit et al., 2011). Lower quality of life, anxiety, depression and lowered self‐esteem emerge in the face of this fatal illness (Antoniou et al., 2020; Cox et al., 2020). Antifibrotic drugs such as pirfenidone and nintedanib may induce progression‐suppressing effects for lung function, exercise tolerance and progression‐free survival for a while (Maher & Strek, 2019). While these drugs may delay the progression of idiopathic pulmonary fibrosis, it is symptom relief that is difficult due to the lack of effective drugs (Pleasants & Tighe, 2019).

Because people with IPF continue to suffer from their symptoms on a daily basis, they reduce their range of activities as their disease progresses losing activities of daily living and independence (Igai, 2019b). In addition, some symptom management (such as using an oxygen tank in public) and the lack of timely and appropriate health care (misdiagnosis, lack of knowledge about IPF) threatened people's dignity (Igai, 2019b).

While Oechsle et al. (2014) also found that the continual struggle of patients with cancer associated symptom management was a threat to their dignity, the question remains about the nuances of how IPF impacts the dignity of patients. In other words, it may not be useful to assume their threats to dignity are the same as for patients with cancer.

The disease is progressive and fatal; therefore, non‐pharmacological care may provide measures for symptom management and quality of life maintenance. Given the nature of IPF progression, a stepwise approach model over time gives a realistic framework for the management of people with IPF which of necessity must be changed with the course of the illness (Richeldi et al., 2017). Initially, the patient is provided with disease‐suppressing medications and patient education. As the disease progresses pulmonary rehabilitation and periods of using oxygen at home increase until finally high‐flow oxygen and hospitalizations are necessary. The patient's end‐of‐life preferences and palliative care should be discussed especially as the disease progresses.

Caring for people with an intractable fatal disease, so they can live in dignity is an important goal and the cornerstone role of palliative care (WHO, 2020). Although the evidence‐based guidelines for diagnosis and management of idiopathic pulmonary fibrosis recommend palliative care (Raghu, et al., 2011), effective palliative care for people with IPF has not been studied. Therefore, there is need to understand and develop palliative care within a context of improving the quality of life for people with idiopathic pulmonary fibrosis.

### 1.1 Background

A systematic narrative literature review about palliative care for those with IPF found that the study designs were mostly qualitative or retrospective studies and were very limited (Igai, 2018). All nine retrospective studies reported that end‐of‐life discussions were less common with patients with IPF than with patients with cancer (41% vs 59%, respectively). Of the two RCT only the Bajwah et al. (2015) study included individualized patient conferences addressing end of life and palliative care needs and reduced symptoms. The RCT by Lindell et al. (2010) reduced quality of life and increased anxiety in patients, although patients reported more support (Igai, 2019a). Surveys of the treatment experiences of people with IPF (Igai, 2019b) have found that people with IPF have difficulty with symptom relief and live with loss of their independence and roles. Researchers described beneficial IPF palliative care models as including: the end‐of‐life discussion, advanced care planning, and symptom management (Raghu & Richeldi, 2017). When their dignity was preserved, people with IPF felt more empowered and generated a more positive self‐image (Griffin‐Heslin, 2005).

Human dignity is an inseparable value of being and has been explicated in both Eastern and Western philosophies and religions (Dan‐Cohen, 2011); human dignity is a cornerstone value in nursing (International Council of Nurses, 2012). Stievano and Tschudin (2019) point out that ethical qualities listed in the nurses' code of ethics such as honesty, kindness, equality and courage are also the qualities of human dignity.

A concept analysis of dignity based on Rodgers's evolutionary method of concept analysis (Rodgers & Knafl, 2000) revealed the relevance of addressing self‐esteem as part of dignity‐centred palliative care (Igai, 2020). Self‐esteem includes components of how one feels about one's self and how others view one; in other words, it is a perception of one's self not necessarily a reality. Not only is self‐esteem an integral part of dignity, but it also plays an important role in the direct and non‐direct impact on chronic disease including symptom severity (Juth et al., 2008).

Therefore, this feasibility study aimed to evaluate the “limited efficacy” and “acceptability” (Bowen et al., 2009) of a dignity‐centred palliative care programme for people with IPF. To evaluate the potential effectiveness of the programme (limited efficacy), we compared the qualitative data of their perceptions of living with IPF with the quantitative data of their self‐esteem, health‐related quality of life, anxiety, depression, dyspnoea, cough and programme satisfaction. A converging of the quantitative and qualitative date was discussed in relationship to the feasibility (limited efficacy and acceptability) of the palliative care programme.

### 1.2 Research questions

Research questions were as follows: (1) Does participation in this programme change the person's perception of living with IPF? (2) Does participation in this programme's intervention result in a change of self‐esteem, health‐related quality of life, anxiety, depression, dyspnoea and cough compared to before the intervention group? and (3) Does the meta‐inference reflecting the integration of the qualitative data of the perception of living with IPF and the quantitative data of self‐esteem, health‐related quality of life, anxiety, depression, dyspnoea, cough and programme satisfaction support the feasibility of the programme?

### 1.3 Conceptual framework for dignity‐centered palliative care

**FIGURE 1:**
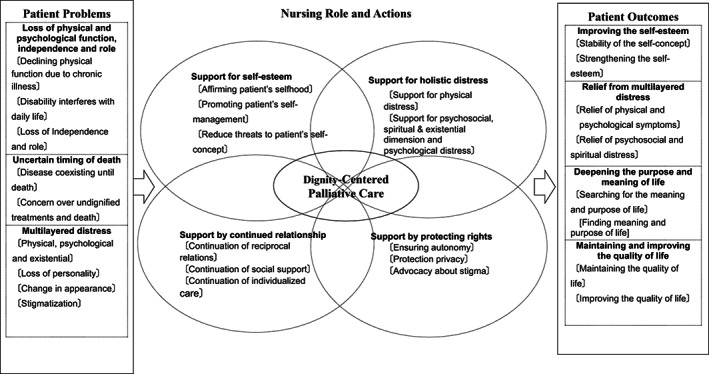
Conceptual framework for dignity‐centred palliative care for people idiopathic pulmonary fibrosis

From the afore‐mentioned concept analysis (Igai, 2020), a conceptual framework was developed that provided “interlinked concepts” for a comprehensive understanding (Jabareen, 2009) (see Figure 1). The conceptual framework for dignity‐centred palliative care included patients' needs, role of the nurse, nursing actions and patient outcomes.

**FIGURE 2:**
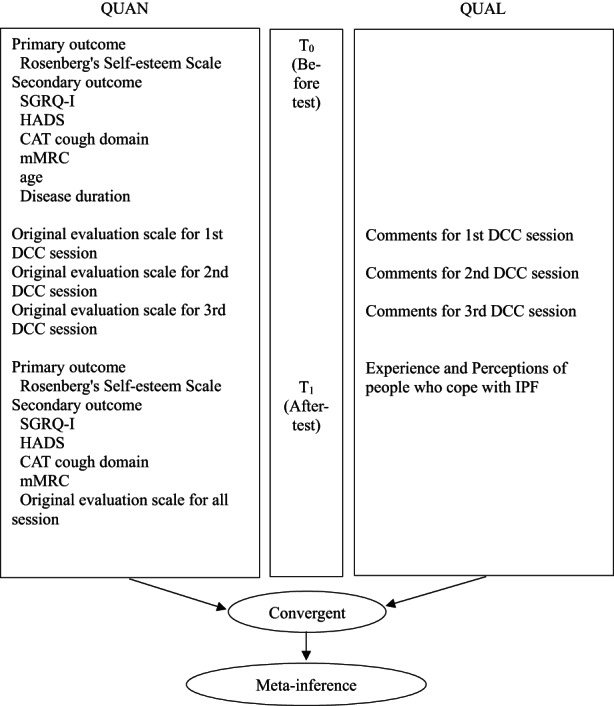
Study diagram of the mixed methods research. CAT, COPD assessment test cough domain; DCC, dignity‐centred care; HADS, Hospital Anxiety and Depression Scale; IPF, idiopathic pulmonary fibrosis; mMRC, Modified Research Council Dyspnoea Scale; QUAL, qualitative dates; QUAN, quantitative dates; SGRQ‐I, IPF‐specific version of the St George's respiratory questionnaire

Patients' needs were as follows: (a) a need for nurses' continued involvement because of lost physical function, (b) maintain psychological capacity and (c) retain autonomy. The nurse' role in dignity‐centred care included four actions: protecting the individual's rights, bolstering self‐esteem, reducing multifaceted distress and supporting continued relationships with family, friends and providers (Igai, 2020) (see Tables 1 and 2). Nursing actions aimed to improve patient's self‐esteem, relieve multi‐faceted distress, assist patient to deepen the purpose and meaning of life, and maintain or improve patient's quality of life. Patient outcomes were: improved self‐esteem, relief of multifaceted distress, deepened purpose and meaning of life and maintenance or improvement in quality of life. Figure 2 displays the conceptual framework of this mixed methods study.

**TABLE 1:** Interventions and rationale of the dignity‐centred palliative care programme for people with idiopathic pulmonary fibrosis

Theme	Location	Rationale of the DCC
Session 1: Self‐management of symptom observation, coping and life review	Outpatient	Bolstering self‐esteem Reducing multi\faceted distress Support by continuing relationship Support while protecting rights
Session 2: Patient education of daily activities and life review	Visiting participant's home	
Session 3: Patient education of living knowledge and life review	Outpatient	

*Note*: Dignity‐centred palliative care (DCC) programme is provided using the booklet. This programme was carried out at the hospital, at home and again at the hospital, three times within 45 days and about 60 minutes for each session.

**TABLE 2:** Content of the dignity‐centred palliative care programme booklet

Sections	Session contents	Session rationale
Section 1	Introduction of dignity‐centered palliative care About human dignity What does dignity‐centered palliative care bring Contents of each session and schedule	Support for holistic distress Support for physical distress Support for psychosocial, spiritual and existential dimension and psychological distress Support by protecting rights Ensuring autonomy Protection privacy Advocacy about stigma
Introduction		
What is dignity‐centered care?		
Contents of program		
Section 2 Disease and treatment	About the Idiopathic Pulmonary Fibrosis Diagnosis, inspections, symptom, natural history, risk factor, acceptable the care by the healthcare provider	
Session 3 Symptom observation and coping	What are the symptoms that require observation? Preventing acute exacerbation and discovering it at an early period How to judge consultation of emergency room Methods of checked their dyspnoea and temperature	
Session 4 Daily activities	Why do daily activities slowly? What is hypoxia? When is exertion? Daily activities and breathing techniques Methods of daily activities Avoiding better living behavior Recommended daily activities Prevention of falls	
Session 5 Knowledge of life	Practices of physical exercises and abdominal breathing Method of panic control Side effects and self‐management of antifibrotic drugs: pirfenidone and nintedanib About your lifestyle habits Drug therapy monitoring Sleeping and Resting Infection prevention Living environment No smoking Be careful with fire during oxygen therapy use Advice regarding healthy eating Oxygen cannula and skin care	
Session 6 Life review	What is life review? Methods of the life review for this program Theme of life reviews How have you faced adversities in life? What do you do to love yourself and others? What brings you joy? What do you think about life? What do you appreciate in your life?	Support for self‐esteem Affirming patient's selfhood] Promoting patient's self‐management Reduce threats to patient's self‐concept
Session 7 Review of today's session	Review of today's session Consultation and use of social resources, information Matters to inform the home healthcare providers Your record—about the process of the condition	Support by continued relationship Continuation of reciprocal relations Continuation of social support Continuation of individualized care

*Note*: Dignity‐centred palliative care programme was provided using this booklet for people with IPF. The programme is carried out: at a hospital, at home, at hospital, three times within 45 days and within 60 minutes for each session.

## 2 METHODS

### 2.1 Study design

#### 2.1.1 Convergent design of the qualitative‐driven mixed methods research study

A single arm and non‐randomized, convergent design of the qualitative‐driven mixed method research was used to evaluate the applicability of this programme (STROBE; von Elm et al., 2007; File S1). Bharmal et al. (2018) explained that mixed methods research on rare and incurable diseases is of high value in understanding the burden of disease and the evaluation of the best management, treatment and care for people living with incurable diseases. Thus, qualitative data were analysed to give descriptions of the experiences and the perceptions of day‐to‐day life of people with IPF in the programme intervention. Quantitative data provided measurements of self‐esteem, quality of life and symptoms. Joint display provided a visual means to represent the results of the qualitative and the quantitative data along with a new inference referred to as a meta‐inference, one that could not be identified by only individual data analysis (Guetterman et al., 2015; Molina‐Azorin & Fetters, 2018). Therefore, in order to describe the reality of dignity for people with IPF, it was considered appropriate to use a convergent design of the qualitative‐driven mixed methods study. The programme was evaluated for feasibility because dignity‐centred palliative care for people with IPF needed unique consideration (Bowen et al., 2009).

### 2.2 Participants

A sample size of 32 patients was determined based on a study of improved self‐esteem in schizophrenic patients through nursing interventions (Seo et al., 2007). Mean values before and after the intervention were compared. In that study, a relative change of self‐esteem was considered statistically significant at the 5% level (two‐sided) with 80% power. The magnitude size of the variability alpha was estimated to have a standard deviation of 4.63 before the intervention and 2.97 to 3.50 after the intervention. In addition, the alpha error was calculated assuming that both sides were 5% and the detection power 1‐β was 80%, with 28 participants. The dropout rate of 15% was based on the incidence of acute exacerbations (Japan Respiratory Society, 2016) and 10% reported by Lindell et al. (2010).

Purposive sampling included the following: (a) stable outpatients who met the diagnostic criteria set out in the joint American Thoracic Society (ATS), European Respiratory Society (ERS), Japanese Respiratory Society (JRS) and the Latin American Thoracic Society (ALAT) consensus statement for the diagnosis of IPF (Raghu, et al., 2011); (b) patients diagnosed as having one of the stages (1–4) in the classification of IPF disease severity and (c) who could endure 60‐minute sessions. Exclusion criteria included patients with idiopathic pulmonary fibrosis associated with lung cancer, declining cognitive function, or unable to read and write in Japanese.

**FIGURE 3:**
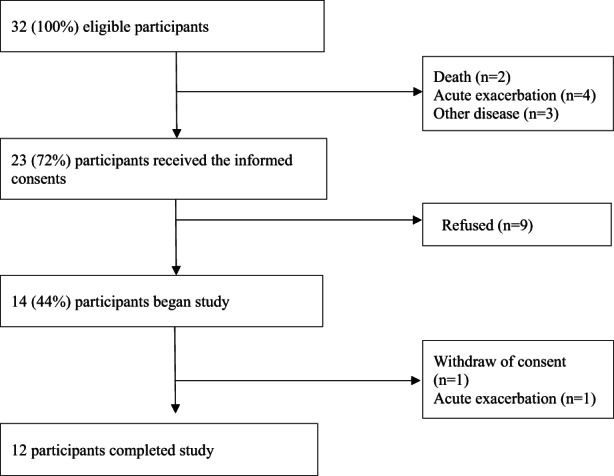
Study flowchart

For recruiting participants, Japanese Respiratory Society board certified physicians distributed recruitment leaflets, to eligible patients while in their examination room. These physicians were trained to avoid coercion and to guarantee the patient's right to decline this study with no adverse repercussions. They referred interested eligible patients to the researcher who further explained the study and confirmed their consent. All participants provided written informed consent. Of the 32 recruited between 18 April 2018–18 March 2019, 14 gave consent and 12 completed this study. One participant had withdrawn consent and the other one was hospitalized with an acute exacerbation. See flow chart Figure 3.

The study was approved by the St. Luke's International University (No. A17‐101) and by the research ethics committees of each of the five hospitals participating in the research before the research commenced.

### 2.3 Dignity‐centred care programme as a palliative care intervention

Dignity‐centred palliative care programme included three interactive modules. It was delivered individually for each participant by the same certified nurse specialist in chronic care. This occurred over a 4–8 weeks: once at the patient's home and twice in a private room of the hospital's outpatient clinic. The modules included: (a) symptom management, (b) enhancing daily activities and (c) life reviews to surface their meaning of living. Life review promotes the maintenance or improvement of the self‐esteem by the reflecting on one's unique life (Haight & Burnside, 1993) and has been reported to improve the quality of life and spiritual well‐being in people with malignant tumours (Ando et al., 2010; Mok et al., 2012). Dr. Viktor E. Frankl's writings (Breitbart et al., 2015) influenced the life review focus about the meaning of life (see Tables 1 and 2 for details).

### 2.4 Outcome measures for feasibility

Quantitative and qualitative programme evaluations for limited efficacy and acceptability (Bowen et al., 2009) occurred after each session and at the end for the programme as a whole. Bowen et al. (2009) describe limited efficacy as answering the question: does using the new programme or model provide some indication of success with the target population, even in the study setting? Key aspects of this study to determine limited efficacy (Bowen, et al., 2009) of the interventions flowing from the dignity‐centred palliative care programme were quantitative changes in self‐esteem respiratory quality of life, anxiety, depression, dyspnoea, cough and programme satisfaction from baseline to after the intervention (see Tables 1 and 2).

### 2.5 Measures for limited efficacy

Self‐esteem was measured using the reliable (internal consistency, test–retest and Cronbach's coefficient M = 0.81) and valid (predictive, criterion) Japanese version of the Rosenberg's Self‐esteem Scale (Beeber et al., 2007; Sakurai, 2000; Sinclair et al., 2010). It is a Likert Scale, with ten items answered on a four‐point scale with responses ranging from *strongly disagree* (1) to *strongly agree* (4). The higher score indicates better self‐esteem. (see Table 3).

**TABLE 3:** Data source, measurements and timing

Data source	Measurements	n	Timing				
			T_0_				T_1_
Quantitative data		*N* = 12					
Primary outcome							
Self‐esteem	Rosenberg's Self‐esteem Scale		×				×
Secondary outcome							
HRQOL	SGRQ‐I		×				×
Anxiety and depression	HADS		×				×
Dyspnoea at the rest	mMRC		×				×
Dyspnoea of the exercise	Modified Borg Scale		×				×
Cough	COPD assessment test cough domain		×				×
Evaluation of the session	Original questionnaire			×	×	×	
Evaluation of the programme	Original questionnaire						×
Qualitative data		*N* = 12					
Perception of IPF	Semi‐structured interviewing						×
Comments	Interviewing			×	×	×	

*Note*: HRQOL = health‐related quality of life; SGRQ‐I = IPF‐specific version of the St George's Respiratory Questionnaire (Yorke et al., 2010); HADS = Hospital Anxiety and Depression Scale (Zigmond et al., 1993); Modified Borg Dyspnoea Scale (Borg, 1982); Modified MRC = Modified Research Council Dyspnoea Scale (Celli et al., 2004); COPD assessment test cough domain (Nishiyama et al., 2017); Original questionnaire = researcher developed for this study.

Respiratory quality of life was measured using the widely used IPF‐specific version of the St George's Respiratory Questionnaire Japanese version‐I (SGRQ‐I). The SGRQ‐I is a 34‐item disease‐specific questionnaire that has been construct validated for IPF by Yorke et al. (2010) and asks questions with respect to three different domains: (a) symptoms, Cronbach's alpha = 0.62 (respiratory: cough, sputum, breathing difficult, wheezing attacks [6 items]); (b) activity, Cronbach's alpha = 0.80 (limited by dyspnoea [10 items]); and (c) impact, Cronbach's alpha = 0.85 (overall life disturbance [18 items]). Each domain is scored from 1 to 100; the higher score indicates a worse quality of life (see Table 3).

Anxiety and depression were measured by the Hospital Depression and Anxiety Scale (HADS‐T) Japanese version (Matsudaira et al., 2009), which includes the HADS‐Anxiety (HADS‐A) with a Cronbach's alpha of 0.81and HADS‐Depression (HADS‐D) with a Cronbach's alpha of 0.76. The HADS‐T is a 14‐item questionnaire, which assesses symptoms of depression and anxiety independent of physical symptoms. HADS‐T scores of 20 or higher indicate either depression or anxiety. A score of eight or more on the HADS‐Anxiety (HADS‐A) is considered anxiety. A score of 11 or more on the HADS‐Depression (HADS‐D) is considered depression (Zigmond et al., 1993). (see Table 3).

Dyspnoea was measured by two reliable and validated scales. The modified Borg Scale (mBS) is a 10‐point scale with numbers and words denoting 1 = *no problem breathing* to 10 = *very difficult to breath*. Comparisons of peak expiratory flow rate, mBS and Sao_2_ percentages demonstrated criterion validity (Kendrick et al., 2000). The modified Medical Research Council dyspnoea (mMRC) Scale (Celli et al., 2004) is scored: 0 = *only with strenuous exercise* to 4 *= too breathless to leave the house*. Higher score indicates worse dyspnoea. Test–retest reliability was confirmed by interclass correlations coefficients of 0.82 at baseline and 0.82 at follow‐up (Mahler et al., 2009) (see Table 3).

Cough was evaluated using the valid and reliable (Cronbach's alpha 0.89) COPD assessment test (CAT) cough domain (Grufstedt et al., 2018; Nishiyama et al., 2017; Tsuda et al., 2012). This self‐report questionnaire consists of eight items scored on a 5‐point Likert Scale. Higher scores indicate a worse cough (see Table 3).

### 2.6 Measures for acceptability

Acceptability, using the Bowen et al.' model (2009), was assured if the participants responded that the programme was suitable, satisfying or attractive. Session and programme evaluation data were derived from two 5‐item original questionnaires. One was for after each session, and the second was once for the programme as a whole. They are scored on a 4‐point scale (4 = *yes*, 3 = s*omewhat*, 2 = *less than somewhat*, 1 = *no*). Examples of items are as follows: “This session made my daily life better,” “This session relieved my suffering from my illness,” and “This session reminds me of the meaning of my life.” The programme as a whole had similar questions. Psychometrics on the questionnaire were not performed (see Table 3).

### 2.7 Qualitative data

Comments about each session's acceptability were gathered during 30–60 minutes semi‐structured interviews regarding experiences and perceptions of people who cope with IPF. These were conducted by the same certified nurse specialist in chronic care, after the completion of the intervention. The interview guide had structured three questions: (1) How do you feel about living with IPF after completing this programme? (2) How do you feel about this programme in terms of alleviating your physical and psychological distress? (3) How do you feel about this programme in terms of helping you strengthen the meaning of life and the purpose of life? The interviews were audio‐recorded with the consent of the participants (see Table 3).

### 2.8 Statistical analyses

Qualitative data from the comments and semi‐structured interviews were analysed using inductive thematic content analysis. Common words were clustered as codes, which were abstracted into categories. We confirmed the interpretation of the data with the participants and received supervision from a gerontological nursing expert to improve the credibility of the findings.

Shapiro–Wilk test for normality indicated that the CAT cough domain (statistics = 0.73, *p* = .002) and HADS‐A (statistics = 0.76, *p* = .003) before the intervention, and HADS‐D (statistics = 0.85, *p* = .04) after the intervention was rejected. Therefore, the quantitative data were analysed using Wilcoxon signed rank sum test, and chi‐square non‐parametric tests. The significance level was set for a probability less than .05.

Content analysis outcomes regarding living with IPF and self‐esteem outcomes were integrated after the intervention of this programme. Qualitative and quantitative data relationships and the meta‐inference were described using a joint display. This study was registered with Japan's UMIN Clinical Trials Registry (ID: UMIN000031861).

## 3 RESULTS

Baseline characteristics of participants are shown in Table 4. Participants were 12 males (92%) 71 to 84 years old.

**TABLE 4:** Baseline characteristics

Variable	IPF (*N* = 12)
Female/male (*n*/%)	1 (8.3)/11 (91.7)
Age (years)	77.3 ± 4.6
The classification of disease severity of IPF (I/ II/ III/ IV)	3/1/5/3
%FVC (%)	77.4 ± 15.2
Smoking status (pack‐year)	38.0 ± 28.0
Received the antifibrotic drugs therapy (*n*/%)	11 (91.7)
Duration of illness (months)	80.4 ± 48.5
Used of LTOT (exercise/24 hr, *n*)	3/3
Months of LTOT use (mean ± SD)	15.3 ± 26.3

*Note*: Data are expresses as mean ± SD.

Abbreviations: FVC, forced vital capacity; IPF, idiopathic pulmonary fibrosis; LTOT, long‐term oxygen therapy.

### 3.1 Quantitative results

#### 3.1.1 Limited efficacy

##### Rosenberg Self‐esteem Scale (primary outcome)

The Rosenberg Self‐esteem Scale at baseline was a median of 28.0 (IQR 23.25–32.25), and after the intervention, the median was 30.0 (IQR 24.0–33.5) with no statistically significant difference (Z = −1.31, *p* = .19). Between baseline and postintervention, the self‐esteem scores increased in eight participants, remained unchanged in one participant and decreased in three participants. Results of the primary outcomes are presented in Table 5.

**TABLE 5:** Outcomes and changed in symptoms at baseline and after the intervention of dignity‐centred care

	Range	Baseline (T_0_) *N* = 12					After intervention (T_1_) *N* = 12						
		*Mdn*	*Q*	*Mean*	*SD*	Cronbach's *α*	*Mdn*	*Q*	*Mean*	*SD*	Cronbach's *α*	Z*	*p*‐ Value
Primary outcome													
Rosenberg Self‐esteem Scale^a^	10–40	28.00	23.25, 32.25	27.58	5.82	0.79	30.00	24.00, 33.50	29.25	5.28	0.77	−1.31	0.19
Secondary outcomes													
SGRQ‐I^b^													
Symptom	0–100	54.19	36.66, 72.03	53.18	22.07	NA	37.34	29.53, 62.49	42.01	20.19	NA	−2.20	0.03
Activity	0–100	67.97	57.75, 89.23	70.09	20.45	NA	73.45	67.25, 89.23	71.91	21.97	NA	−0.56	0.58
Impact	0–100	46.83	25.85, 56.13	46.23	22.11	NA	33.75	23.72, 47.73	38.90	23.17	NA	−1.41	0.16
Total scores	0–100	56.30	41.12, 61.06	54.19	17.97	NA	44.54	37.21, 62.00	48.66	20.43	NA	−1.57	0.12
HADS^c^													
Anxiety	0–21	13.00	12.00, 14.00	13.25	2.05	0.48	14.00	11.25, 14.75	13.00	1.89	0.36	−0.16	0.88
Depression	0–21	15.00	11.00, 18.00	14.25	3.82	0.76	12.50	11.25, 16.25	12.67	3.12	0.58	−0.47	0.64
Total score	0–42	28.00	24.25, 31.00	27.50	4.21	0.66	26.00	24.25, 31.75	25.67	4.10	0.61	−0.40	0.69
Modified Borg Scale^d^	0–10	3.00	1.25, b5.00	3.21	2.31	NA	2.50	0.63, b4.75	2.83	2.43	NA	−1.91	0.06
mMRC^e^	0–4	2.00	1.00, b2.00	1.58	0.79	NA	2.00	1.00, b2.00	1.58	0.79	NA	−0.00	1.00
CAT (cough)^f^	0–5	2.00	1.00, b2.00	1.75	0.83	NA	1.50	1.00, b2.00	1.67	1.37	NA	−0.26	0.79

Abbreviations: *Mdn*, median; NA, not available; *Q*, quartile; Z*, Wilcoxon Signed Rank Test.

^a^ Japanese Version of Rosenberg Self‐esteem Scale (Sakurai, 2000).

^b^ SGRQ‐I, Japanese Version of IPF‐specific Version of St George's Respiratory Questionnaire (Yorke et al., 2010).

^c^ HADS, Japanese Version of the Hospital Anxiety and Depression Scale (Zigmond et al., 1993).

^d^ Modified Borg Scale = Modified Borg Dyspnoea Scale (Borg, 1982).

^e^ Modified MRC = Modified Medical Research Council Dyspnoea Scale (Celli et al., 2004).

^f^ CAT = COPD Assessment Test cough domain (Nishiyama et al., 2017).

##### St. George respiratory quality of life

Of the three SGRQ‐I Japanese version subscales (symptoms, impact and activity), only the symptoms subscale showed a statistically significant improvement between baseline and after the intervention (Z = −2.20, *p* = .03) (see Table 5).

##### HADS‐A, HADS‐D and HADS‐T (anxiety and depression)

There were no statistically significant differences for HADS‐A, HADS‐D and HADS‐T between baseline and after intervention (Z = −0.16, *p* = .88; Z = −0.47, *p* = .64; Z = −0.40, *p* = .69, respectively). All 12 participants scored 20 (HADS‐T) or more at baseline and after the intervention indicating anxiety and depression (see Table 5).

##### Dyspnoea and cough

Dyspnoea on exercise measured by the modified Borg Scale and modified MRC revealed no statistically significant difference from baseline and after the intervention (Z = −1.91, *p* = .06; Z = 0·00, p = 1·00). Cough measured using the CAT cough domain showed no statistically significant difference before and after the intervention (Z = − 0.26, *p* = .79) (see Table 5).

##### Session and programme satisfaction

Sessions were rated from a median of 16 to 18 out of 20. The programme median as a whole was 17 (IQR 12.5–19.5). Notable items scoring a maximum of 4 were as follows: relieves my suffering, makes my life better and more aware of the meaning of life. (see Table 6).

**TABLE 6:** Evaluation of session and programme results

Contents		Programme						T_1_	
		1st session		2nd session		3rd session			
		Outpatient		Visiting home		Outpatient			
		Contents: Symptom management and life review		Contents: Activity of daily life and life review		Contents: Knowledge of daily life and life review		All programme	
		*Mdn*	*Q*	*Mdn*	*Q*	*Mdn*	*Q*	*Mdn*	*Q*
Total	1–20	16.0	13.5, 18.5	18.0	15.0, 19.0	15.0	15.0, 20.0	17.0	12.5, 19.5
1. This programme enhances your feelings of cherishing yourself	1–4	3.0	2.5, 4.0	3.0	3.0, 4.0	3.0	3.0, 4.0	3.0	3.0, 4.0
2. This programme will make my life better	1–4	3.0	3.0, 4.0	4.0	3.0, 4.0	3.5	3.0, 4.0	4.0	3.0, 4.0
3. This programme relieves the suffering of my illness	1–4	4.0	3.0, 4.0	3.0	3.0, 4.0	3.5	3.0, 4.0	3.0	3.0, 4.0
4. This programme makes me aware of the meaning of life	1–4	3.0	2.5, 4.0	4.0	3.0, 4.0	3.0	3.0, 4.0	3.0	2.3, 4.0
5. This programme enriches my life	1–4	3.0	2.0, 3.5	3.0	3.0, 4.0	3.5	3.0, 4.0	3.0	3.0, 4.0

Abbreviations: *Mdn*, median; *Q*, quartile.

### 3.2 Qualitative results

#### 3.2.1 Acceptability

Extracted from the semi‐structured interviews were eight categories (Table 7) that indicated participants' perceptions of living with IPF involved a change in their self‐esteem. Participants were stratified into two groups based on their self‐esteem scores. Those who had maintained or increased self‐esteem after the intervention (*n* = 9) revealed the following categories: (a) getting information about illness; (b) changing thinking and behaviour to cope with physical and psychological distress; (c) supported by interactive interchange; and (d) holding a positive attitude for living with IPF. Participants whose self‐esteem had declined (*n* = 3) revealed the following categories: (e) uncertainty about the progress of idiopathic pulmonary fibrosis; (f) uncertainty about their prognosis; (g) continued self‐management; and (h) life review is useless.

**TABLE 7:** Extracted categories and sub‐categories from the qualitative data

Categories	Main sub‐categories	Raw data
Getting information about illness	I was able to understand IPF by getting information and knowledge Think how to live with IPF	“Learning about IPF through this program, I was able to accept living with IPF”
Changing thinking and behaviour to cope with physical and psychological distress	Changing the way of thinking even if you cannot move Protect my mind and body while changing daily activities	“I was able to understand how to breathe, and it was very good my mentally”
Supported by interactive interchange	Two‐way interaction brings a sense of stability I'm glad you listened to my story	“I was able to learn a lot and ask questions about IPF”
Holding positive attitude for living with IPF	Establishing an attitude to live with IPF Realize my life that I have lived Accept of my illness has not changed	“I was able to take a head‐on attitude toward this illness”
Uncertainty about the progress of idiopathic pulmonary fibrosis	No place to learn about IPF IPF is an unknown disease Life will be restricted by oxygen in the future	“I didn't understand my illness”
Uncertain prognosis	Need more drug development than care I will never die of IPF	“I want you to spread the treatment drug faster than mental support”
Continued self‐management	Opportunity to gain knowledge to help self‐management Repeat self‐learning using a special booklet	“This program reinforces self‐management”
Do not need the life review	There is no point in looking back on my life The meaning of life is useless	“I didn't really understand the need for a life review”

### 3.3 Integrated results of the qualitative and quantitative data analyses

The converged data results shown a joint display (Table 8) are arranged in a single figure for comparison (Creswell, 2015). In the joint display, the meta‐inferences are described, which resulted from the integrated qualitative and quantitative data.

**TABLE 8:** Joint display of the acceptance of living with IPF and self‐esteem change after dignity‐centred palliative care programme intervention

Participants (age, gender)		Quantitative data						Meta‐inference
	Qualitative data	Primary outcome	Secondary outcome		Attributes			
	The acceptance of living with IPF							
Self‐esteem (range: 10–40)	Modified Borg Scale^a^	CAT cough domain^a^	IPF severity	HOT (Y/N)	Months of disease^b^
77, M	【Getting information about illness】 【Changing thinking and behaviour to cope with physical and psychological distress】 【Supported by interactive interchange】 【Holding attitude for living with IPF】	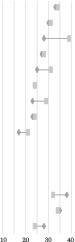	0·5	−2·0	I	N	62	The nine participants who maintained and increased their self‐esteem deepened their understanding of IPF, and they had to change their lifestyle behaviours that needed correction to deal with life problems. One also acknowledged that his dyspnoea was alleviated due to changes in his daily activities, and he was willing to take the attitude of living with IPF. By contrast, the three who perceived uncertain prognosis of IPF, and found life review useless had declined self‐esteem. Of the three, two participants underwent HOT only during exertion. Three participants were in IPF severity classification III. In meta‐inference, the perceptions regarding IPF were related in changing in self‐esteem.
84, M			−1·0	−1·0	I	N	19	
83, M			±0	+2·0	II	N	34	
72, F			±0	+1·0	I	N	63	
83, M			±0	−1·0	IV	Y	92	
72, M			−1·0	±0	IV	Y	123	
77, M			−0·5	±0	III	N	71	
76, M			±0	±0	III	Y	94	
71, M			±0	±0	IV	Y	51	
82, M	【Uncertainty about the progress of idiopathic pulmonary fibrosis】【Uncertain prognosis】 【Continued self‐management】 【Do not need the life review】		−1·0	±0	III	E	216	
78, M			±0	+1·0	III	E	68	
72, M			−1·5	−1·0	III	N	72	

*Note*: Modified Borg Scale (Borg, 1992). Graph of self‐esteem: Before = ♦; After = 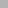.

Abbreviations: CAT cough, COPD assessment test cough domain; E, home oxygen therapy during exercise; F, female; HOT, home oxygen therapy; IPF, idiopathic pulmonary fibrosis; M, male; N, no; Y, yes.

^a^ Amount of change.

^b^ Month.

Participants were divided two groups: (a) those who maintained or increased their self‐esteem (*n* = 9) and (b) those who experienced a decrease, and there was no statistically significant difference before and after the intervention (*n* = 3). The nine participants who maintained or increased their Rosenberg Self‐esteem Scale scores had increased from baseline of zero to 11 points. This group talked about: (a) how this programme helped them understand IPF better, (b) they understood the lifestyle behaviours that needed to be modified, (c) how they changed their lifestyle behaviours, (d) how the life review helped them feel supported by the life they had lived and (e) that they had a more positive attitude of living with IPF. By contrast, of the three participants with lower self‐esteem in this programme, one was aware of dyspnoea during exertion and was thinking about the need for home oxygen therapy and two were on home oxygen therapy only during times of exertion. They talked about (a) not being able to look ahead due to the uncertainty of the course of the IPF, (b) continuing their own self‐management and (e) their belief that life review was not necessary.

## 4 DISCUSSION

This dignity‐centred palliative care programme was developed in order to prevent the dignity of people with IPF from being threatened, due to clinicians' lack of understanding of this rare disease. It is the first to report on the feasibility of an IPF dignity‐centred palliative care programme consisting of patient education and life reviews. The mixed methods study produced a rich data set from which to examine the feasibility of limited efficacy and acceptability (Bowen et al., 2009) of the palliative care programme to promote dignity‐centred palliative care. Self‐esteem resulted as a key feasibility marker for those living with IPF.

### 4.1 Participant characteristics

The participants in this study are similar to the population of people with IPF. Factors of older age, males and smoking history are similar to the IPF risk factor reports from the Japanese Respiratory Society (2016), the American Thoracic Society (2000), the report of the prevalence in UK (Strongman et al., 2018) and the epidemiological study in Japan by Natsuizaka et al. (2014).

### 4.2 Feasibility

#### 4.2.1 Limited efficacy

##### Self‐esteem

Although nine of the 12 had increased self‐esteem scores after the intervention, the difference between baseline and after intervention was not statistically significant. Furthermore, with the implementation of home oxygen therapy, various challenges are expected to arise in terms of both the daily living activities and their feelings about themselves. This group is considered to have difficulty maintaining or increasing self‐esteem. In the meta‐analysis of people with chronic obstructive pulmonary disease (COPD), self‐esteem was a positive factor influencing the dyspnoea and symptom domains of the SGRQ (Cannon et al., 2018). Therefore, given the positive changes in self‐esteem this programme intervention has limited efficacy.

##### SGRQ‐I Japanese version (symptoms, activity and impact)

In our study, the symptoms (severity and frequency of respiratory symptoms) in the SGRQ‐I Japanese version revealed statistically significant improvement between the baseline and after intervention in the SGRQ‐I Japanese version thus establishing programme limited efficacy. Similar to our study, the participants in the Duck et al. (2015) programme for people with IPF may have found coping strategies and behavioural changes that resulted in symptom relief. It is possible that participants' behavioural changes in activities of daily living such as breathing exercises helped to alleviate symptoms. A randomized controlled trial (Vainshelboim, 2016) found consistent bi‐weekly breathing and aerobic exercises led to improved symptoms.

##### HADS‐A, HADS‐D and HADS‐T (depression and anxiety)

All participants entered the programme with high HADS depression and anxiety scores, unlike only 25.9% of Korean participants with IPF who were considered depressed and 21.4% who were anxious (Lee et al., 2017). There were no statistically significant differences in anxiety, depression and the total score of anxiety and depression between baseline and after intervention. Janssen et al. (2020) also found no improvements in anxiety or depression in their examination of referrals for palliative care in their RCT. Lindell et al. (2010) reported that anxiety tended to increase in the intervention group in a randomized controlled trial of disease‐management education. Longer programmes and continuity of emotional support are critical programme components and should be considered to increase programme efficacy.

##### Borg, MRC and CAT (dyspnoea and cough)

For dyspnoea, there was a non‐significant trend of improvement between baseline and after intervention on the modified Borg Scale and MRC. There was no statistically significant change in the CAT, which measured cough. A systematic review and meta‐analysis found that pulmonary and aerobic exercises significantly increased lung capacity and breathing (Hanada et al., 2020). A more long‐term programme with a strong breathing exercise component would provide a stronger limited efficacy for IPF palliative care.

##### Programme satisfaction

The programme satisfaction scores were positive and confirmed the programme acceptability. However, the programme was not a light‐hearted or entertaining type of programme. It would have required introspection and facing facts. Given that component, the scores were very encouraging.

##### Perceptions of the living with IPF

Based on the knowledge of IPF gained through this programme, the eight participants who increased in self‐esteem were able to change their thoughts and living behaviours and cope with their physical and psychological distress. It is probably that one of the things that supported this coping was the interactive support between the participant and the certified nurse specialist in chronic care as the nurse researcher of this study. Incorporating the meaning and purpose of the life as a theme of the life review brought a new awareness to the participants. A pilot study, of the life review on the theme of meaning and purpose in people with malignancies, reported that this intervention was effective in improving their quality of life, especially their existential distress (Mok et al., 2012). The finding from this study also suggests that the life review, which focuses on the meaning and purpose of the life, assists people with IPF to build on their past and to discover how they will live with IPF in the future.

By contrast those who had lower self‐esteem, thought the life review had no value suggesting the two factors might be related. The participants expressed they did not know about IPF and how it progressed. They also spoke of the life review in this programme as being unhelpful. The Westerhof and Slatman (2019) meta‐analysis of life review therapy noted that depressed individuals recalled negative life events, which would reinforce the depressive thinking and feeling patterns or as revealed in this programme, reminding participants of the unpleasant memories that they had unconsciously repressed. Therefore, the efficacy of this programme may be more appropriate for those who have accepted their illness and then offer a modified version developed based on participants level of depression and disease acceptance.

### 4.3 Integration of perceptions living with IPF and changes in self‐esteem

Examining limited efficacy and acceptability by integrating perceptions of living with IPF and changes in self‐esteem provided a detailed picture. Extracted from the qualitative data for nine of the 12 participants were the categories: (a) getting information about illness; (b) changing thinking and behaviour to cope with physical and psychological distress; (c) supported by interactive interchange; and (d) holding positive attitude for living with IPF. Participants described that their self‐esteem was maintained or increased, daily living behaviours were adapted to their changing body, and they had a positive attitude of the living with IPF as a result of their life review. Ando et al. (2010) found that people can easily lose their identity due to severe illness, but the life story constructed by the individual gives them meaning, confirms their identity and self‐continuity, and enhances the completeness of their life, which supports our research. IPF is a physically and psychologically demanding disease. The four participants whose self‐esteem scores increased by four points or more had low IPF severity levels of I to II, and home oxygen therapy was not as yet necessary.

The stage of the four participants whose self‐esteem increased by only one point tended to be severe. They used home oxygen therapy and their disease had progressed. However, they had already accepted their IPF diagnoses before the intervention, and their acceptance did not change after the completion of this programme. For those who originally had accepted their IPF diagnoses, their change in self‐esteem scores was not large. Therefore, it is necessary to understand the acceptance of IPF including their narratives, not only the change in scores.

The characteristics of the participants with low self‐esteem were those who used home oxygen therapy only for exercise or those who did not used home oxygen therapy yet, although they noticed increasing dyspnoea. Khor et al. (2017) reported that for those with interstitial lung disease, the initiation of the home oxygen therapy symbolized the end stage of this disease. The psychological impact of using home oxygen therapy is statistically significant and the approaching need for home oxygen therapy during exertion may have lowered their self‐esteem.

Aspects of this programme, such as meaning of life review, were considered too advanced for those who were using home oxygen therapy only during the exercise or expected to use the home oxygen therapy in the near future and were prone to self‐image fluctuations. Therefore, it is important to know how they perceive living with the disease before the intervention. It is also important to support people's identity by validating their deeper sense of self as opposed to just roles they have played such as father or worker.

### 4.4 Meta‐inference

Self‐esteem was maintained or increased through this programme for the nine participants who tended to: (a) have a better understanding of IPF, (b) transform their thoughts and life behaviours and (c) have a positive attitude of living with IPF. By contrast, self‐esteem decreased for the three participants who tended to perceive life review as not being necessary because of the uncertainty of the course of IPF. Acceptance of living with IPF is involved in a positive change of self‐esteem. Home oxygen therapy as a symbol of health decline is a possible threat to self‐esteem.

## 5 LIMITATIONS

Limitations of the study were that participants had dyspnoea, and thus, the intervention had time constraints; there was no control group and there may have been selection bias due to purposive sampling. The small sample size (due to a rare intractable disease) was less of a limitation as this was a feasibility study. Unfortunately, acute exacerbations, deaths and dropouts (62%) during the period leading up to eligible patients receiving an explanation greatly increased the estimated dropout (15%). Of the 14 participants who originally consented to be in this study, 12 participants were able to complete this programme. One person dropped out during the study period due to an acute exacerbation and one dropped out for unknown reasons. Reasons for dropouts (excluding exacerbations and death) in this study were not obtained, and in future research, this would be important to include to reduce bias. Therefore, research on IPF is challenging not only because it is rare but also because the mortality is high. The data from the 12 that completed the palliative dignity‐centred care programme are of great value that may contribute to the palliative care of other lung malignancies.

Most programmes for life review are at least 10 weeks long (Westerhof & Slatman, 2019); therefore, this programme may have been too brief. The interventionist and the evaluator were the same nurse, which could have introduced bias. However, because the interventionist was a certified specialist nurse in chronic care, this may have positively affected these results by providing participants continuity, exceptional practice and expertise regarding IPF. Finally, psychometric test should be applied to the two original questionnaires to strengthen programme evaluation. However, all other quantitative questionnaires in this study had validity and reliability and the qualitative study followed a systemic method of contents analysis. The design of the mixed method research increased the validity and reliability.

## 6 CONCLUSION

Integrating the qualitative and quantitative data, about daily activities, symptoms in daily life and positive attitudes living with IPF from this programme provided a richer understanding that supported the feasibility a dignity‐centred palliative care programme. Nine participants who had accepted living with IPF maintained or increased their self‐esteem. However, three participants whose IPF experienced uncertainty and were not yet able to find meaning in the life reviews and had decreased self‐esteem. Meta‐inference results indicated that positive acceptance of IPF maintained and increased self‐esteem; therefore, this programme may be more useful for those who have accepted their IPF. Regardless of self‐esteem, participants generally found the programme acceptable.

## AUTHOR CONTRIBUTIONS

YI conceive the study design, recruited the participants, provided the nursing palliative care program and wrote the first draft of the manuscript. YI and SP analysed, interpreted the research data and edited and revised the manuscript. All authors contributed to, and reviewed the writing of the manuscript and approved its submission.

## CONFLICT OF INTEREST

The authors declared that they have no conflict of interests.

## CLINICAL TRIAL REGISTRATION NUMBER

This study is registered with the UMIN clinical trials registry (ID: UMIN000031861).

## Data Availability

The datasets generated and analysed during the current study are available from the corresponding author on reasonable request.
